# Neurophysiological features associated with suicidal risk in dementias

**DOI:** 10.1192/j.eurpsy.2021.2193

**Published:** 2021-08-13

**Authors:** I. Mudrenko, A. Yurchenko

**Affiliations:** Department Of Neurosurgery And Neurology, Sumy State University, Sumy, Ukraine

**Keywords:** electroencephalographic correlates, suicidal risk, dementias.

## Abstract

**Introduction:**

The bioelectrical activity of the brain of suicidals has specific features.

**Objectives:**

Investigate neurophysiological features associated with high suicidal risk (SR) in dementias.

**Methods:**

An electroencephalographic study of brain was performed in 66 patients with dementia, of which 33 (with high SR) were included in the main group, the other 33 (with low SR) – in the control group.

**Results:**

SR correlates include an increase in the spectral density and amplitude (in μV) of the α-rhythm in the right central (C4) (109.4) – in the main group, compared with (64.5) – in the control; in the temporal areas (T4) (132.2) - in the main group, (70.0) – in the control group (p<0.001). The predominance of the spectral density of the slow θ-rhythm over the entire surface of the brain (p<0.001) and δ-rhythm in the projection of Fp2 (82.3) – in the main and (116.1) – in the control groups (p<0.001), F3 (54.80) and (68.1), respectively, (p<0.05), F4 (52.4) and (67.3), respectively, (p<0.01), C4 (52.0) and (62.0), respectively (p<0.05), P3 (44.4) and (58.9), respectively, (p<0.01), O1 (67.6) and (89.41), (p<0.001), O2 (68.5) and (85.8), respectively (p<0.001) are a predictor of low SR in dementias.

**Conclusions:**

With the progression of changes in the brain in dementias SR decreases. In the initial phases of the dementing process with a relatively preserved functional capacity of the brain SR is high.

**Disclosure:**

No significant relationships.

